# Assessing Adherence to Healthy Dietary Habits Through the Urinary Food Metabolome: Results From a European Two-Center Study

**DOI:** 10.3389/fnut.2022.880770

**Published:** 2022-06-09

**Authors:** Pol Castellano-Escuder, Raúl González-Domínguez, Marie-France Vaillant, Patricia Casas-Agustench, Nicole Hidalgo-Liberona, Núria Estanyol-Torres, Thomas Wilson, Manfred Beckmann, Amanda J. Lloyd, Marion Oberli, Christophe Moinard, Christophe Pison, Jean-Christian Borel, Marie Joyeux-Faure, Mariette Sicard, Svetlana Artemova, Hugo Terrisse, Paul Dancer, John Draper, Alex Sánchez-Pla, Cristina Andres-Lacueva

**Affiliations:** ^1^Nutrimetabolomics Laboratory, Department of Nutrition, Food Sciences and Gastronomy, XIA, INSA, Faculty of Pharmacy and Food Sciences, University of Barcelona, Barcelona, Spain; ^2^CIBER Fragilidad y Envejecimiento Saludable (CIBERfes), Instituto de Salud Carlos III, Madrid, Spain; ^3^Statistics and Bioinformatics Research Group, Department of Genetics, Microbiology and Statistics, University of Barcelona, Barcelona, Spain; ^4^Laboratory of Fundamental and Applied Bioenergetics, Inserm1055, Grenoble, France; ^5^Service Hospitalier Universitaire Pneumologie Physiologie, CHU Grenoble Alpes, Grenoble, France; ^6^Institute of Biological, Environmental and Rural Sciences, Aberystwyth University, Aberystwyth, United Kingdom; ^7^Groupe SEB, Lyon, France; ^8^Université Grenoble Alpes, Grenoble, France; ^9^IC@dom, Investigation Clinique à domicile, Meylan, France; ^10^CHU Grenoble Alpes, CIC-IT, Grenoble, France; ^11^TIMC-MESP Laboratory, University of Grenoble Alpes, Grenoble, France

**Keywords:** Alternative Healthy Eating Index (AHEI-2010), metabolomics, diet quality, microbiota, dietary assessment

## Abstract

**Background:**

Diet is one of the most important modifiable lifestyle factors in human health and in chronic disease prevention. Thus, accurate dietary assessment is essential for reliably evaluating adherence to healthy habits.

**Objectives:**

The aim of this study was to identify urinary metabolites that could serve as robust biomarkers of diet quality, as assessed through the Alternative Healthy Eating Index (AHEI-2010).

**Design:**

We set up two-center samples of 160 healthy volunteers, aged between 25 and 50, living as a couple or family, with repeated urine sampling and dietary assessment at baseline, and 6 and 12 months over a year. Urine samples were subjected to large-scale metabolomics analysis for comprehensive quantitative characterization of the food-related metabolome. Then, lasso regularized regression analysis and limma univariate analysis were applied to identify those metabolites associated with the AHEI-2010, and to investigate the reproducibility of these associations over time.

**Results:**

Several polyphenol microbial metabolites were found to be positively associated with the AHEI-2010 score; urinary enterolactone glucuronide showed a reproducible association at the three study time points [false discovery rate (FDR): 0.016, 0.014, 0.016]. Furthermore, other associations were found between the AHEI-2010 and various metabolites related to the intake of coffee, red meat and fish, whereas other polyphenol phase II metabolites were associated with higher AHEI-2010 scores at one of the three time points investigated (FDR < 0.05 or β ≠ 0).

**Conclusion:**

We have demonstrated that urinary metabolites, and particularly microbiota-derived metabolites, could serve as reliable indicators of adherence to healthy dietary habits.

**Clinical Trail Registration:**

www.ClinicalTrials.gov, Identifier: NCT03169088.

## Introduction

Diet (with exercise) is recognized nowadays as one of the most important modifiable lifestyle factors in human health and in the prevention of chronic diseases (1). Moreover, a suboptimal diet quality has been described as a leading cause of death and a well-established risk factor for many diseases, such as coronary heart disease, type 2 diabetes and multiple types of cancer (2–4). In nutritional research, dietary assessment is often conducted via self-reporting methods, such as food frequency questionnaires (FFQs) and dietary recalls (5). Furthermore, several scores have been validated in associating food intake data with overall dietary patterns and with adherence to healthy dietary habits (6). Among these scoring methods, the Alternative Healthy Eating Index (AHEI-2010) has been validated in different populations as one of the most powerful indexes in predicting the risk of chronic disease related to dietary behavior (7).

The growing field of metabolomics has enabled in-depth exploration of the food-related metabolome and the identification of potential food intake biomarkers (8). Biomarkers enable accurate and objective dietary assessment, which can mitigate the inherent misreporting errors of traditional self-reported methods, and thus provide a closer and more reliable overview of the interplay between diet and health. Nevertheless, although numerous studies have previously investigated the association between circulating metabolites and the consumption of particular food groups, only a few have focused on the identification of biomarkers of overall healthy dietary patterns. In this respect, various authors have recently addressed the identification of candidate metabolomic markers of the AHEI-2010 in blood serum/plasma samples from different populations (9–12). The majority of the metabolites that were identified as being significantly associated with the AHEI-2010 score were lipids, and to a lesser extent amino acids and other endogenous metabolite classes, most of which can be regarded as biomarkers of effect rather than real biomarkers of exposure (13). This bias toward the endogenous metabolome could be largely due to the investigation of blood as the biological matrix under study, since this biofluid contains considerably lower concentrations of food-related metabolites than urine, which is the preferred sample for reflecting the human metabolism at the end of the Absorption, Distribution, Metabolism and Excretion (ADME) process (8). Furthermore, it should be noted that all these studies relied on the application of untargeted metabolomics approaches, which usually hinders the detection of minor exogenous metabolites derived from diet and other lifestyle habits. As an alternative, the application of multi-targeted urinary metabolomics platforms has demonstrated increased sensitivity, reproducibility and coverage for comprehensive and quantitative analysis of the food metabolome (14), which could provide new insights into the characteristic metabolomics signatures associated with adherence to healthy dietary habits.

In this study, we aimed to identify urinary metabolites associated with the AHEI-2010 score, which could thus serve as biomarkers of adherence to healthy dietary patterns. For this purpose, a large-scale quantitative metabolomics platform was employed for comprehensive analysis of almost 350 urinary food-related and microbiota-derived metabolites (15, 16). To validate the robustness of these associations, repeated urine sampling and dietary assessment were performed at three study time points over the course of a year in a multi-center study.

## Materials and Methods

### Study Design

A total of 160 free-living healthy subjects were enrolled in a population of healthy volunteers followed for 1 year (ClinicalTrials.gov Identifier: NCT03169088). The recruitment was conducted between 2016 and 2018 in two different study centers: Université Grenoble Alpes (France, *n* = 100) and Aberystwyth University (Wales, United Kingdom, *n* = 60). The inclusion criteria were as follows: healthy people living as a couple or family and involved in organizing and cooking meals in the household, ages ranging from 25 to 50, non-smokers, body mass index (BMI) ≥25, <30 (representative of the French and Welsh populations), and no chronic use of medications that may influence metabolism, gut microbiota or dietary behavior. All the participants were advised to adhere to healthy dietary habits in accordance with general nutritional recommendations: “The Eatwell Guide” 2016 for the UK population; https://www.gov.uk/government/publications/the-eatwell-guide, and Mangerbouger, Programme National Nutrition Sante, https://www.mangerbouger.fr/ the “Programme National Nutrition Santé” for the French population; https://solidarites-sante.gouv.fr/prevention-en-sante/preserver-sa-sante/le-programme-national-nutrition-sante/article/programme-national-nutrition-sante-pnns-professionnels, respectively). Over a year, anthropometric variables (i.e., weight, BMI) and vital signs were assessed at baseline and at 12 months (Supplementary Table S1), to confirm that the subjects were healthy. Furthermore, dietary intake data and urine samples were collected at each time point, as detailed below. The study was performed in accordance with the principles contained in the Declaration of Helsinki and the recommendations for Good Clinical Practice. The French Ethics Committees (CPP Sud-Est V and ANSM) and the Aberystwyth University Ethics Committee approved the study protocol, and all the participants provided written informed consent.

### Dietary Assessment

Dietary intake was assessed at each study point over a year (i.e., M0, M6, and M12) using validated country-specific food frequency questionnaires (FFQs), which were composed of 118 and 135 food items for the French and UK populations (17, 18), respectively. The FFQ data were then employed to calculate the updated AHEI-2010 score as described by Chiuve et al. (7). The AHEI-2010 consists of 11 components, namely vegetables, fruits, whole grains, sugar-sweetened beverages and fruit juices, nuts and legumes, red/processed meats, trans fats, long-chain n-3 fatty acids, polyunsaturated fatty acids, sodium, and alcohol. Among these components, higher intakes of sugar-sweetened beverages and fruit juices, red/processed meats, trans fats and sodium are associated with lower scores, while vegetables, fruits, whole grains, nuts and legumes, long-chain n-3 fatty acids and polyunsaturated fatty acids contribute positively to the AHEI-2010. With regard to alcohol, the highest score is assigned to moderate, and the lowest score to high, alcohol consumers. The score for each of the 11 components ranged from 0 (lowest) to 10 (highest) points, so that the overall AHEI-2010 score ranged from 0 to 110 points, with higher scores denoting a healthier diet. In this study, a slight modification of the original AHEI-2010 method was applied because French food composition tables do not include trans fatty acid analysis. Accordingly, the AHEI-2010 score was calculated on the basis of the 10 remaining components (Supplementary Table S2), as previously reported (19).

### Urine Collection and Preparation

The 160 subjects enrolled in the population-based cohort first provided morning void urine samples at M0, M6, and M12 over a year. At each time point, a total of nine spot urine samples were collected on three random days during a week in three different weeks over a five-week period. Samples were collected at home using vacuum transfer technology (20), stored at −20°C in the participants' domestic fridges until the end of each sampling period (i.e., M0, M6, and M12), and then transported to the research facilities to be made acellular and then for long-term storage at −80°C (20).

The nine spot urine samples collected within each sampling period were normalized by refractive index and pooled to create a single sample per person that reflected the individuals' habitual diet at M0, M6, and M12 (21). For this purpose, the urine samples were thawed at 4°C, vigorously vortexed and centrifuged at 10,000 ×g for 10 min at 4°C. Then, 200 μl of the supernatants were transferred to a refractometer to record the specific gravity (OPTi Digital Handheld Refractometer, Bellingham & Stanley, UK). Using these data, the specific gravity correction factors were calculated as a fold change of each urine specific gravity to the lowest urinary specific gravity measured for that participant at each time point. In accordance with these correction factors, urine samples were aliquoted and supplemented with ultra-pure water to make a total volume of 500 μl, thereby ensuring that all samples had the same refractive index. Finally, equal volumes of the nine diluted urine samples per time point were pooled for metabolomics analysis.

### Metabolomics Analysis of Urine Samples

Urine samples were subjected to large-scale metabolomics analysis for comprehensive quantification of almost 350 food metabolites and their host and microbial derivatives, following the methodology developed by González-Domínguez et al. (15, 16). To this end, solid-phase extraction with Oasis® HLB extraction plates (Waters, Milford, MA, USA) was applied in order to extract and pre-concentrate urinary polyphenols and other food-related compounds, as well as their biotransformed metabolites (i.e., phase I/II and microbiota derivatives). Furthermore, urine samples were also subjected to tenfold dilution to analyse highly concentrated and polar metabolites. A set of internal standards was added to the samples for quantification and quality control assessment (15). Analyses were then carried out by ultra-high performance liquid chromatography coupled to tandem mass spectrometry (UHPLC-MS/MS) using the operating conditions described elsewhere (15, 16). Metabolomics results were normalized in reference to the urinary refractive index to account for inter-individual differences in hydration status and micturition frequency.

### Quality Control Assessment

Metabolomics data were subjected to quality control assessment prior to statistical analysis using a standardized protocol developed in-house and implemented in the POMA R/Bioconductor package (22). For each of the three data blocks (i.e., M0, M6, and M12), an independent quality control assessment and data preprocessing were performed as follows. First, data were log-transformed and Pareto-scaled, and Euclidean distances to the group centroid were computed to remove outliers from the data matrix (±3×IQR). Then, the concentrations of metabolites known to be influenced by pre-analytical factors (e.g., improper handling/storage of samples) were inspected to check for the absence of abnormal values (±3×IQR). Finally, the coefficients of variation for peak areas, retention times and peak widths of the internal standards were computed for evaluating the analytical reproducibility (<15% for peak areas and widths, <2% for retention times) (14).

### Statistical Analysis

Least Absolute Shrinkage and Selection Operator (LASSO) regression was first used to select those metabolites with the highest predictive capacity for predicting the AHEI-2010 at each of the three time points (i.e., M0, M6, and M12). In this process, the continuous variable AHEI-2010 was used as the response and the whole metabolomics matrix as predictors. In addition, a limma (linear models for microarray data) univariate approach was employed to complement and validate the results uncovered by LASSO regression. The limma models were adjusted for the study center (i.e., France or UK), and the resulting metabolites were selected according to a false discovery rate (FDR) adjusted *p* < 0.05.

## Results

### Characteristics of the Population-Based Cohort

From the 160 participants initially enrolled in our population of healthy volunteers, several dropouts emerged over a year, including subjects who withdrew from the study or did not provide complete urine samples and/or dietary intake data at all of the three study time points (Supplementary Figure S1). At baseline, the participants' ages ranged between 25 and 50, and the proportion of women was slightly higher than that of men (61%). The calculated AHEI-2010 scores were in the range 51.2–53.8 across the three study time points, in line with the values reported for the normal population in previous studies (7). The AHEI-2010 scores of the study population at the three study time points divided by center and sex are shown in Table 1. Additionally, the AHEI-2010 individual component scores at the three study time points also divided by center and sex are shown in Supplementary Table S3. It is worth noting that women had slightly higher average AHEI-2010 scores than men, and a small increase of 2.6 points was observed over the 1-year period in the overall population.

**Table 1:** AHEI-2010 scores of the study population at the three study time points (M0, M6 and M12), divided by center and sex.

**Time**	**Center**	**Sex**	***n***	**AHEI-2010 score**
M0	Center 1	Men	40	49.9 ± 10.3
		Women	57	52.0 ± 10.7
	Center 2	Men	20	50.8 ± 11.4
		Women	40	51.2 ± 7.1
	**Total M0**		**157**	**51.2 ±9.8**
M6	Center 1	Men	37	51.4 ± 10.6
		Women	54	54.4 ± 12.4
	Center 2	Men	15	52.0 ± 9.8
		Women	29	51.8 ± 8.2
	**Total M6**		**135**	**52.8 ±10.8**
M12	Center 1	Men	31	52.6 ± 10.7
		Women	50	55.2 ± 11.9
	Center 2	Men	15	51.1 ± 9.3
		Women	31	54.0 ± 5.8
	**Total M12**		**127**	**53.8 ±10.1**

*AHEI-2010 scores are presented as mean ± SD*.

### Identification of Urinary Metabolites Associated With the AHEI-2010 Score

A large-scale metabolomics platform was applied for comprehensive and quantitative analysis of the urinary food metabolome, encompassing polyphenols and other food-origin compounds, metabolites derived from the host metabolism (i.e., phase I/II metabolism) and microbiota derivatives (15, 16).

**Figure 1:**
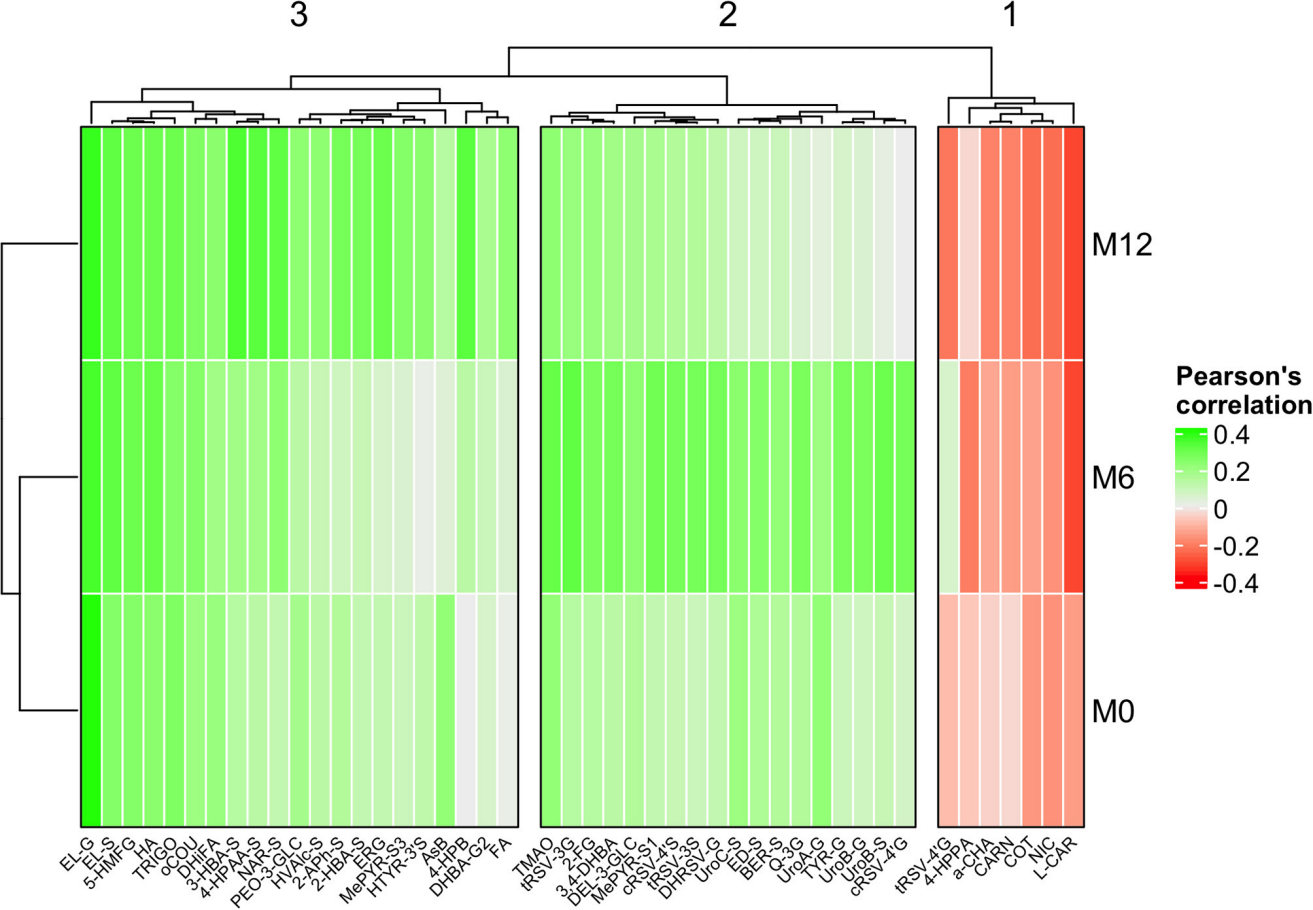
Clustered heatmap showing Pearson's correlation across the three study points between the AHEI-2010 and those metabolites selected by LASSO or limma approaches, respectively. Cluster 1 shows those metabolites that tend to be negatively associated with AHEI-2010 at the three study time points. Clusters 2 and 3 show those metabolites positively associated with AHEI-2010 at each of the three study time points, making a slight distinction between those most strongly associated at time M6 (Cluster 2), and times M0 and M12 (Cluster 3).

The use of LASSO regression enabled us to reduce the number of metabolites considerably from almost 350 features to 45 compounds with predictive capacity at a minimum of one of the three study points. Then, limma univariate analysis was employed to complement and validate the findings from the LASSO multivariate approach. Additionally, Pearson's correlation between the AHEI-2010 and those selected metabolites was also computed. As shown in Table 2, most of the metabolites that were identified as being associated with the AHEI-2010 (Figure 1) were microbiota-derived compounds, including enterolignans, urolithins and various classes of phenolic acids (e.g., hydroxybenzoic, hydroxyphenylacetic and hydroxycinnamic acids) and derivatives (e.g., pyrogallol derivatives). As shown in Figure 1, these microbial metabolites were positively associated with the AHEI-2010 score, but only enterolactone glucuronide was consistently replicated at the three study points investigated here. Similarly, several polyphenol phase II metabolites were also associated with higher AHEI-2010 scores, including stilbenes, furanocoumarins, anthocyanins, flavanones, phenylethanoids and flavonols, which can serve as markers of the consumption of red wine, grapefruit, berries, citrus, olive oil and plant foods, respectively (23). However, none of these associations were replicated at the three time points. A reproducible positive association was observed between urinary 5-(hydroxymethyl-2-furoyl) glycine (5-HMFG) and the AHEI-2010 over a year, which was accompanied by a positive association with 2-furoylglycine (2-FG) at M6 and with trigonelline at M0 and M12, thus providing strong evidence of an association between coffee consumption and healthy dietary habits. Although not corroborated at baseline, a robust association was also found with various metabolites related to red meat (i.e., L-carnitine and carnosine, negative association) and fish (i.e., trimethylamine N-oxide and arsenobetaine, positive association) intake. Finally, a few other metabolites were identified only at M12, including 4-hydroxyproline betaine (i.e., citrus metabolite) and ergothioneine (i.e., mushroom metabolite), which were positively associated with the AHEI-2010, whereas α-chaconine (i.e., potato metabolite) and nicotine-derived compounds (i.e., tobacco metabolites) showed an inverse association (Figure 1).

**Table 2:** Metabolites identified as being associated with the AHEI-2010 score at M0, M6 and M12.

		**M0**			**M6**			**M12**		
		**LASSO (β)**	**LIMMA (logFC)**	***r***	**LASSO (β)**	**LIMMA (logFC)**	***r***	**LASSO (β)**	**LIMMA (logFC)**	***r***
Microbial metabolites
	*Enterolignans*									
	Enterolactone glucuronide	5.6	0.016	0.40	2.3	0.014	0.36	3.5	0.016	0.38
	Enterolactone sulfate			0.25		0.00057	0.32		0.00057	0.32
	Enterodiol sulfate			0.16	0.3		0.25			0.08
	*Urolithins*									
	Urolithin A glucuronide	2.7		0.22			0.20			0.03
	Urolithin B glucuronide			0.08		0.0040	0.27			0.05
	Urolithin B sulfate			0.10	17.5	0.00095	0.30			0.02
	Urolithin C sulfate			0.20	37.0		0.24			0.08
	*Phenolic acids*									
	2-Hydroxybenzoic acid sulfate			0.11			0.10		0.0015	0.28
	3-Hydroxybenzoic acid sulfate			0.14			0.19	1.1	0.016	0.35
	3,4-Dihydroxybenzoic acid			0.13		0.0020	0.28			0.19
	Dihydroxybenzoic acid glucuronide			0.06			0.09	0.1		0.17
	Hippuric acid			0.24		0.013	0.31		0.013	0.30
	4-Hydroxyphenylacetic acid sulfate			0.13			0.19	1.2	0.017	0.33
	o-Coumaric acid			0.20			0.25		0.0012	0.26
	Ferulic acid			0.01			0.05	1.5		0.23
	3-(4-hydroxyphenyl)propionic acid			−0.07	−9.1		−0.21			−0.04
	Dihydroisoferulic acid			0.21			0.19	3.9		0.24
	Methylpyrogallol sulfate			0.11	0.6	0.0067	0.29		0.0096	0.17
	2-Aminophenol sulfate			0.15			0.08	0.8	0.013	0.28
Flavonoids
	*Stilbenes*									
	trans-Resveratrol 3-glucuronide			0.14	3.5	0.0037	0.32			0.21
	trans-Resveratrol 4'-glucuronide			−0.09			0.06	−4.8		−0.22
	cis-Resveratrol 4'-sulfate			0.10	5.2	0.00097	0.28			0.15
	cis-Resveratrol 4'-glucuronide			0.07		0.0021	0.28			0
	trans-Resveratrol 3-sulfate			0.09		0.00097	0.28			0.15
	Dihydroresveratrol 3-glucuronide			0.11	12.1	0.00057	0.28			0.12
	*Furanocoumarins*									
	Bergaptol sulfate			0.15	4.6		0.22			0.09
	*Anthocyanins*									
	Delphinidin 3-glucoside			0.15			0.23	61.8		0.18
	Peonidin 3-glucoside			0.18			0.13	54.1		0.23
	*Flavanones*									
	Naringenin sulfate			0.11			0.23	4.8	0.00082	0.32
	*Phenylethanoids*									
	Hydroxytyrosol 3'-sulfate			0.12			0.01	3.3		0.24
	Homovanillyl alcohol sulfate			0.13			0.10	0.2		0.23
	Tyrosol glucuronide			0.09		0.0042	0.27			0.06
	*Flavonols*									
	Quercetin 3-glucuronide			0.15	7.0	0.0012	0.27			0.05
Coffee
	5-(hydroxymethyl-2-furoyl)glycine (5-HMFG)	2.7		0.25	7.2	0.0050	0.30	2.2	0.0057	0.30
	2-furoylglycine (2-FG)			0.14	0.9	0.0052	0.27			0.21
	Trigonelline	1.0		0.26			0.25	1.6	0.016	0.30
Meat and fish
	L-carnitine			−0.15	−6.8	−0.0047	−0.31	−6.8	−0.0054	−0.31
	Carnosine			−0.04			−0.15	−1.8		−0.20
	Trimethylamine N-oxide			0.22	1.6	0.014	0.31			0.23
	Arsenobetaine			0.22			0.04	2.6		0.14
Others
	Ergothioneine			0.11			0.06	3.9	0.0047	0.30
	4-Hydroxyproline betaine			0			0.13	2.1	0.017	0.33
	α-Chaconine			−0.05			−0.13	−47.1		−0.20
	Nicotine			−0.17			−0.17	−2.4		−0.24
	Cotinine			−0.16			−0.14	−0.3		−0.24

*For the LASSO values, only the non-zero β coefficients are shown, while for the LIMMA values, only those logFC values with an associated FDR < 0.05 are shown. Pearson's correlation (r) between the selected metabolites and the AHEI-2010 are also presented in the table*.

## Discussion

Metabolomics has demonstrated great potential for discovering candidate biomarkers reflecting the consumption of particular foods and adherence to complex dietary patterns (8, 24). However, adequate replication studies to evaluate the robustness of these potential markers are still required, especially considering the high intra- and inter-individual variability of the human metabolome (25). In this study, we aimed to identify urinary metabolites associated with healthy dietary habits, as assessed through the AHEI-2010, and to investigate the reproducibility of these associations over time at three study points over 1 year in a free-living, population-based cohort. To obtain a reliable snapshot of the individuals' habitual diet, we employed the sampling method proposed by Beckmann et al. (21) based on the assumption that the collection and pooling of various spot urine samples should provide a single representative sample of the dietary habits adhered to during the sampling time frame. Furthermore, repeated sampling was conducted at 0, 6 and 12 months (M0, M6 and M12) over a year to investigate the reproducibility of the metabolites analyzed here. In this respect, it has been reported that three urine samples collected within 1 year are sufficient for the measurement of long-term exposure status in epidemiological studies (26).

The most robust result was the positive association between the AHEI-2010 scores and urinary levels of enterolactone glucuronide, which was consistently replicated at M0, M6 and M12, using both LASSO regression and limma univariate analysis (Table 2). Enterolactone is the main microbial-derived metabolite of dietary lignans, a subclass of polyphenols widely distributed in plant foods such as fruits, vegetables, wholegrains, legumes and nuts (27). Lignans are well known to possess anti-inflammatory and antioxidant properties due to their phytoestrogen nature; various epidemiological studies have shown that high circulating concentrations of enterolactone are associated with a decreased risk of cardiovascular diseases (28), several cancers (29), neurodegenerative disorders (30) and other chronic diseases. Notably, Whitton et al. (31) reported that urinary enterolactone was positively correlated with various dietary quality scores in a multi-ethnic Asian population, including the AHEI-2010, the alternate Mediterranean Diet (aMED) and the Dietary Approaches to Stop Hypertension (DASH). In line with these results, Wellington et al. (32) also reported increased levels of urinary enterolactone glucuronide after an intervention with a Prudent diet. In the present study, enterolactone glucuronide showed a robust association over time with the AHEI-2010 score across the three sampling periods, and it was detected at concentrations that are easily measurable by using state-of-the-art analytical techniques (0.006–27.6 μmol L^−1^). Accordingly, it could be regarded that this metabolite is a reliable and robust biomarker for assessing the healthiness of a diet, in agreement with the guidelines for the validation of food intake biomarkers (33).

In this respect, we also observed a positive association between the AHEI-2010 score and many other polyphenol microbiota derivatives, including enterolignans, urolithins and phenolic acids (Table 2). However, none of these associations were replicated at the three time points investigated. Considering their microbial origin, this variability could be mainly due to changes in the gut microbiota composition over the study year, but also to other phenotypic (e.g., weight changes) and environmental (e.g., changes in dietary habits) factors (34). In particular, it should be noted the existence of inter-individual variability factors related to the incapacity of some individuals to produce specific microbiota derivatives, i.e., the so-called “metabotypes.” This is of particular importance for urolithin production, for which three metabotypes have been described (35), and could be, at least in part, behind the variability reported in the present study. Altogether, our results suggest that microbial-derived metabolites could be better indicators of healthy dietary habits than the corresponding parent polyphenol species. This might be largely due to the poor absorption and extensive metabolization of dietary polyphenols, which makes microbial metabolites the predominant species in circulation, although mounting evidence indicates that microbiota derivatives can also participate in the biological effects traditionally attributed to polyphenols (36).

Aside from the microbiota-related metabolites discussed above, LASSO regression revealed a reproducible association of 5-(hydroxymethyl-2-furoyl)glycine (5-HMFG) with higher AHEI-2010 scores over time, as well as a positive association with 2-furoylglycine (2-FG) at M6. Furan metabolites are intermediate products of the Maillard reactions that are produced in foods during heating processes (e.g., roasting, sterilization). After consumption, furans undergo conversion to 2-furoic acid and 5-hydroxymethylfuroic acid, then conjugation with glycine to form 2-FG and 5-HMFG, and finally excretion *via* urine (37). Thus, glycinated furans have previously been proposed as candidate intake biomarkers for different heat-processed food products, such as dried fruits (38, 39) and coffee (40, 41). In our metabolomics data set, Pearson correlation analyses showed a strong and significant correlation between the two furoylglycine species and other urinary metabolites (*r* > 0.30, Supplementary Table S4), including various methylxanthine alkaloids (e.g., caffeine), diketopiperazines (e.g., cyclo-leucyl-proline) and trigonelline, which are wellknown markers of coffee consumption (42). This therefore suggests that coffee is the most plausible dietary source of 5-HMFG and 2-FG in our population-based cohort. Furthermore, we observed a significant association between trigonelline levels and the AHEI-2010 score at M0 and M12 (Table 2), thus providing robust evidence of the association between coffee consumption and adherence to a healthy diet. In line with our results, recent meta-analyses have related habitual coffee intake with positive health outcomes and with lower incidence of type 2 diabetes, various liver diseases and cancer types, as well as with reduced all-cause mortality (43). However, it is worth noting that coffee is not amongst the food items that are employed for calculating the AHEI-2010 score (7), which reinforces the added value of measuring objective biomarkers in addition to using traditional dietary assessment self-reported methods in order to truly elucidate the impact of diet on health.

Although not corroborated at baseline, we found a strong inverse association between L-carnitine and the AHEI-2010 score at M6 and M12, together with a negative association with carnosine at M12. This could mirror a deleterious effect of red and processed meat consumption on health, since increased urinary levels of these metabolites have been repeatedly reported in habitual consumers of these food products (44). Conversely, various metabolites reflecting the intake of fish and/or shellfish showed associations with the AHEI-2010 in the opposite direction, namely trimethylamine N-oxide at M6 and arsenobetaine at M12 (Table 2). In line with our results, Akbaraly et al. (9) reported a robust association between AHEI-2010 scores and circulating concentrations of omega-3 polyunsaturated fatty acids (positive association) and saturated fatty acids (negative association), which can be mainly found in fish and meat products, respectively (8). In another study, various fish-related metabolites were identified as the most discriminant predictors for distinguishing high from low scores for different dietary patterns, with two of them (i.e., docosahexaenoic acid and hydroxy-3-carboxy-4-methyl-5-propyl-2-furanpropanoate) being shared between the AHEI-2010, the aMED and the Healthy Eating Index (9). These associations found between red and processed meats and fish metabolites with the AHEI-2010 in healthy subjects propose a stage prior to the results found in epidemiological data, which suggest that regular consumption of red and processed meats is closely related to an increased risk of developing cardiovascular diseases and cancer (45), whereas fish intake may contribute to risk reduction for a range of health outcomes (46).

Finally, LASSO regression also evidenced consistent associations between the AHEI-2010 score and other candidate food intake biomarkers defined in the Food-Biomarker Ontology (24), but only at one of the three sampling points investigated. These findings were in turn replicated to a large extent using complementary limma-based univariate analysis (Table 2). In particular, a positive association was found with various red wine- (i.e., trans-resveratrol 3-glucuronide, cis-resveratrol 4′-sulfate, dihydroresveratrol 3-glucuronide) and grapefruit-derived (i.e., bergaptol sulfate) metabolites at M6, as well as with other metabolites related to the intake of citrus (i.e., naringenin sulfate, 4-hydroxyproline betaine), olive oil (i.e., hydroxytyrosol 3′-sulfate, homovanillyl alcohol sulfate), mushrooms (i.e., ergothioneine) and berries (i.e., peonidin 3-glucoside, delphinidin 3-glucoside) at M12. Although fruits, olive oil and moderate red wine consumption represent some pivotal components of many healthy dietary patterns, including the AHEI-2010 (7), the lack of a reproducible association over time with the AHEI-2010 scores was not particularly surprising, because the intake of these foods might be influenced by seasonality factors (note that time points M0 and M12 are slightly different to time point M6 in the clusters in Figure 1). Indeed, these specific food items (e.g., citrus, berries) are included within more complex components of the dietary quality scores (e.g., fruits) (7), so that high scores can be obtained even with low intake of these particular foods if the criteria are met for the other components. Similarly, we also found robust associations between several metabolites related to potato intake (i.e., α-chaconine) and smoking (i.e., nicotine, cotinine) with lower AHEI-2010 scores, but again only at M12. Potatoes are the only vegetables that do not contribute positively to the AHEI-2010 score (7), because their consumption has not been associated with lower chronic disease risk in epidemiologic studies (47) and is linked to increased incidence of diabetes (48). Furthermore, it should be noted that French fries/chips were the main form in which potatoes were consumed in our population-based cohort [ca. 70% of total potato consumption according to the self-reported dietary intake data (unpublished data)]. Similarly, previous studies have reported that dietary patterns characterized by a high consumption of French fries/chips are associated with increased cardiometabolic risk and mortality caused by cardiovascular diseases (49), in line with the inverse association observed in our study between the AHEI-2010 and urinary α-chaconine. On the other hand, although smoking status is not considered within the AHEI-2010 scoring method and was considered an exclusion factor in this study, various nicotine-related metabolites were also identified as reliable predictors of unhealthy dietary habits (Table 2). In this respect, several studies have demonstrated a higher adherence to unhealthy diets and to energy-dense, nutrient-poor food consumption amongst smokers in different populations (50, 51). Altogether, these results evidence that a range of metabolites related to the intake of particular foods (e.g., citrus, red wine, olive oil, potatoes) and lifestyle habits such as smoking or passive smoking (most likely the source of nicotine-related compounds in this study) are strongly associated with adherence to the AHEI-2010. This therefore demonstrates the crucial need to investigate the inter-individual variability in the bioavailability of these candidate biomarkers of healthy dietary habits.

The main strength of this study was investigating—to the best of our knowledge for the first time—the reproducibility of the associations between urinary metabolites and the AHEI-2010 score over time, which is of utmost importance for addressing individual variability in order to identify reliable and robust candidate biomarkers of healthy dietary habits. For this purpose, we leveraged a large-scale metabolomics platform for comprehensive and quantitative characterization of the urinary food metabolome. Unlike previous studies aimed at identifying AHEI-2010-related metabolites by applying untargeted metabolomics to serum/plasma samples, mainly focused on the endogenous metabolome (i.e., biomarkers of effect) (9–12), the methodology employed here enabled us to gain a deeper insight into the food-related and microbiota-derived metabolome (i.e., biomarkers of exposure). However, some limitations should also be noted. First, as in any observational study based on food intake data, our results could be influenced by measurement errors inherent to the self-reported methods employed for dietary assessment (in this study we could not apply a more accurate method such as the technique of duplicate meal trays due to the cost and logistics of applying this method in our population). Furthermore, the AHEI-2010 scoring method is based on a limited set of predefined food groups and therefore does not provide a complete overview of all the nutritional and lifestyle factors involved in the healthiness of a diet. Therefore, further studies in independent and larger population cohorts are needed to validate the findings uncovered here, as well as to find new and good markers for the AHEI-2010 adherence.

## Conclusions

In conclusion, this study has demonstrated that the urinary food-related metabolome is strongly associated with adherence to healthy dietary habits as assessed through the AHEI-2010. Many of the metabolites identified were microbial-derived compounds, including enterolignans, urolithins and phenolic acids, thus confirming the major role played by the gut microbiota in the interplay between diet and health. Despite observing high variability across the three study points for these microbial species, the enterolactone glucuronide metabolite showed a reproducible association over time. Furthermore, robust associations were found between the AHEI-2010 score and various metabolites reflecting the intake of coffee (i.e., 5-HMFG), red meat (i.e., L-carnitine) and fish (i.e., trimethylamine N-oxide, arsenobetaine), while other food products also showed a robust association at one of the three time points investigated here.

## Data Availability Statement

The original contributions presented in the study are included in the article/Supplementary Material, further inquiries can be directed to the corresponding author/s.

## Ethics Statement

The studies involving human participants were reviewed and approved by the French Ethics Committees (CPP Sud-Est V and ANSM) and the Aberystwyth University Ethics Committee approved the study protocol, and all the participants provided written informed consent. The patients/participants provided their written informed consent to participate in this study. Written informed consent was not obtained from the individual(s) for the publication of any potentially identifiable images or data included in this article.

## Author Contributions

CM, CP, PD, JD, and CA-L designed the research. PC-E, RG-D, M-FV, PC-A, NH-L, NE-T, TW, MB, AL, MO, J-CB, MJ-F, MS, and SA conducted the research. PC-E, TW, HT, and AS-P analyzed the data. PC-E and RG-D wrote the paper. CA-L and AS-P had primary responsibility for the final content. All authors read and approved the final manuscript.

## Funding

This work was supported by the EIT Health Innovation by Design Project COOK2HEALTH. EIT Health is supported by the European Institute of Innovation and Technology, a body of the European Union and the Spanish Ministry of Economy and Competitiveness (MINECO) together with the Joint Programming Initiative A Healthy Diet for a Healthy Life (PCIN-2014-133; 2015-238), CIBERfes and ISCIII Projects AC19/00096 (co-funded by the FEDER Program from the European Union, A way to make Europe), and the Generalitat de Catalunya's Agency AGAUR (2017 SGR1546). RG-D thanks the Juan de la Cierva Programme from MINECO (FJCI-2015-26590) and CA-L the ICREA Academia Award 2018.

## Conflict of Interest

The authors declare that the research was conducted in the absence of any commercial or financial relationships that could be construed as a potential conflict of interest.

## Publisher's Note

All claims expressed in this article are solely those of the authors and do not necessarily represent those of their affiliated organizations, or those of the publisher, the editors and the reviewers. Any product that may be evaluated in this article, or claim that may be made by its manufacturer, is not guaranteed or endorsed by the publisher.
