# Supplementary material for: Assessing Adherence to Healthy Dietary Habits Through the Urinary Food Metabolome: Results From a European Two-Center Study
Source: Front Nutr. 2022 Jun 9;9:880770. doi: 10.3389/fnut.2022.880770 (PMC9219016; doi:10.3389/fnut.2022.880770)
Supplement: Supplementary file 1 [file Table_1.DOCX]

Supplementary Material

**Supplementary Table S1.** Anthropometric variables and vital signs at baseline (M0) and M12 for French and Welsh populations.

| France Group | Intervention | Control | p | Ni-Nc |
| --- | --- | --- | --- | --- |
| Weight (kg), median [Q1 ; Q3] |  |  |  |  |
| M0 | 78.0 [70.1 ; 83.6] | 78.3 [72.0 ; 86.4] | . | 50 - 50 |
| M12 | 77.7 [69.7 ; 83.8] | 76.5 [71.6 ; 86.5] | 0,485 | 47 - 46 |
| BMI (kg/m2) |  |  |  |  |
| M0 | 27.1 [25.8 ; 28.3] | 27.4 [26.2 ; 29.2] | . | 50 - 50 |
| M12 | 26.8 [25.5 ; 28.5] | 27.0 [26.0 ; 28.3] | 0,602 | 47 - 46 |
| Systolic Blood Pressure (mmHg), median [Q1 ; Q3] |  |  |  |  |
| M0 | 118.0 [110.0 ; 125.0] | 118.0 [109.0 ; 124.0] | . | 50 - 50 |
| M12 | 114.0 [109.5 ; 122.5] | 115.5 [107.0 ; 122.0] | 0,779 | 48 - 46 |
| Diastolic Blood Pressure (mmHg), median [Q1 ; Q3] |  |  |  |  |
| M0 | 70.0 [66.0 ; 74.0] | 68.0 [62.0 ; 71.0] | . | 50 - 50 |
| M12 | 66.0 [62.0 ; 73.0] | 66.0 [62.0 ; 72.0] | 0,501 | 48 - 46 |
| Heart rate (bpm), median [Q1 ; Q3] |  |  |  |  |
| M0 | 65.5 [58.0 ; 71.0] | 63.0 [57.0 ; 67.0] | . | 50 - 50 |
| M12 | 67.0 [63.0 ; 71.0] | 65.0 [59.0 ; 70.0] | 0,288 | 48 - 46 |
| Respiratory rate (bpm), median [Q1 ; Q3] |  |  |  |  |
| M0 | 20.0 [18.0 ; 20.0] | 18.0 [16.0 ; 18.0] | . | 14 - 24 |
| M12 | 14.0 [13.0 ; 16.0] | 14.0 [13.0 ; 16.0] | 0,348 | 47 - 44 |

| Wales Group | Intervention | Control | p | Ni-Nc |
| --- | --- | --- | --- | --- |
| Weight (kg), median [Q1 ; Q3] |  |  |  |  |
| M0 | 79.3 [70.2 ; 84.5] | 77.3 [71.4 ; 88.6] | . | 30 - 30 |
| M12 | 78.8 [68.6 ; 84.8] | 76.4 [70.2 ; 86.6] | 0,997 | 26 - 27 |
| BMI (kg/m2) |  |  |  |  |
| M0 | 27.2 [26.1 ; 28.7] | 27.0 [25.8 ; 29.2] | . | 30 - 30 |
| M12 | 27.6 [25.2 ; 28.7] | 26.8 [25.8 ; 29.9] | 0,817 | 26 - 27 |
| Systolic Blood Pressure (mmHg), median [Q1 ; Q3] |  |  |  |  |
| M0 | 128.0 [120.0 ; 136.0] | 122.5 [115.0 ; 134.0] | . | 30 - 30 |
| M12 | 120.5 [112.0 ; 127.0] | 122.0 [108.0 ; 130.0] | 0,414 | 26 - 27 |
| Diastolic Blood Pressure (mmHg), median [Q1 ; Q3] |  |  |  |  |
| M0 | 76.5 [70.0 ; 85.0] | 72.0 [69.0 ; 81.0] | . | 30 - 30 |
| M12 | 75.5 [65.0 ; 83.0] | 72.0 [69.0 ; 78.0] | 0,283 | 26 - 27 |
| Heart rate (bpm), median [Q1 ; Q3] |  |  |  |  |
| M0 | 67.0 [61.0 ; 70.0] | 73.0 [69.0 ; 79.0] | . | 30 - 30 |
| M12 | 63.5 [59.0 ; 70.0] | 69.0 [63.0 ; 74.0] | 0,593 | 26 - 27 |
| Respiratory rate (bpm), median [Q1 ; Q3] |  |  |  |  |
| M0 | 19.0 [16.0 ; 22.0] | 17.0 [16.0 ; 19.0] | . | 30 - 30 |
| M12 | 18.0 [15.0 ; 20.0] | 16.0 [14.0 ; 19.0] | 0,098 | 25 - 27 |

**Supplementary Table S2.** AHEI-2010 individual components associated with their nutritional groups/categories.

| AHEI-2010 component | Nutritional group |
| --- | --- |
| A1 | Vegetables |
| A2 | Fruits |
| A3 | Whole grains |
| A4 | Sugar sweetened drinks and fruit juice |
| A5 | Nuts and legumes |
| A6 | Red and processed meats |
| A7 | Long-chain omega-3 fatty acids |
| A8 | Polyunsaturated fatty acids (PUFA) |
| A9 | Sodium |
| A10 | Alcohol |

**Supplementary Table S3.** AHEI-2010 individual component scores presented as mean ± standard deviation at the three study time points. The individual components nutritional groups are described in the Table S2.

| Time | Center | Sex | *n* | A1 | A2 | A3 | A4 | A5 | A6 | A7 | A8 | A9 | A10 |
| --- | --- | --- | --- | --- | --- | --- | --- | --- | --- | --- | --- | --- | --- |
| M0 | Center 1 | Men | 40 | 6,4 ± 2,3 | 6,2 ± 3,3 | 0,8 ± 1,3 | 5 ± 4 | 2,9 ± 2,3 | 3,7 ± 2,6 | 8,8 ± 1,9 | 3,6 ± 1,6 | 5,1 ± 3,4 | 7,5 ± 2,8 |
|  |  | Women | 57 | 6,8 ± 2,6 | 6,4 ± 2,9 | 0,9 ± 0,9 | 5,5 ± 4 | 3,2 ± 2,9 | 4,2 ± 2,7 | 8,4 ± 2 | 4,5 ± 2,1 | 5,7 ± 3,4 | 6,4 ± 2,9 |
|  | Center 2 | Men | 20 | 8,3 ± 2,6 | 5,8 ± 3,2 | 2,3 ± 1,9 | 2,8 ± 4 | 6 ± 3,4 | 3,9 ± 3,1 | 4,8 ± 2,1 | 4,6 ± 1,8 | 5,5 ± 3,8 | 7 ± 3,3 |
|  |  | Women | 40 | 8,5 ± 2 | 6,6 ± 2,7 | 2,9 ± 2,1 | 3,6 ± 4,1 | 3,8 ± 3,2 | 4,6 ± 2,4 | 4,6 ± 2,4 | 4,6 ± 1,3 | 5,2 ± 3,2 | 7 ± 2,9 |
| M6 | Center 1 | Men | 37 | 6,1 ± 2,5 | 5,5 ± 2,7 | 0,8 ± 0,9 | 6,4 ± 3,2 | 3,1 ± 2,6 | 4,6 ± 2,8 | 8,2 ± 2,3 | 3,6 ± 1,8 | 5,7 ± 3,4 | 7,4 ± 2,8 |
|  |  | Women | 54 | 6,7 ± 2,7 | 6 ± 2,9 | 1 ± 1,1 | 5,7 ± 4 | 4,2 ± 3,2 | 4,7 ± 2,5 | 8,4 ± 2,2 | 5,3 ± 2,8 | 6,2 ± 2,9 | 6,3 ± 2,8 |
|  | Center 2 | Men | 15 | 8 ± 2,4 | 5,5 ± 3,8 | 3,3 ± 2,7 | 2,9 ± 3,8 | 5,9 ± 3,6 | 4,5 ± 2,6 | 4,4 ± 2,1 | 5,1 ± 1,9 | 5 ± 3,4 | 7,6 ± 3,2 |
|  |  | Women | 29 | 8,4 ± 2,2 | 6 ± 2,8 | 2,7 ± 1,8 | 4,4 ± 4,1 | 4,5 ± 3,5 | 4,9 ± 2,6 | 4,2 ± 2,1 | 4,7 ± 1,4 | 5,5 ± 3,2 | 6,6 ± 2,7 |
| M12 | Center 1 | Men | 31 | 5,9 ± 2,6 | 5,4 ± 3,1 | 0,6 ± 0,6 | 6,1 ± 3,6 | 3,3 ± 2,7 | 4,3 ± 2,8 | 8,6 ± 2 | 4,2 ± 2,1 | 6,2 ± 3,2 | 8 ± 2,5 |
|  |  | Women | 50 | 6,9 ± 2,8 | 6,1 ± 2,9 | 1,1 ± 1,4 | 6,1 ± 3,8 | 3,9 ± 3 | 4,7 ± 2,4 | 8,3 ± 2,4 | 5,1 ± 2,4 | 6,2 ± 3 | 6,7 ± 2,8 |
|  | Center 2 | Men | 15 | 7,3 ± 2,4 | 4,7 ± 3,4 | 2,5 ± 1,6 | 3,7 ± 3,9 | 5,9 ± 4,1 | 4,2 ± 2,6 | 3,9 ± 2,4 | 4,6 ± 1 | 6,2 ± 3,4 | 7,9 ± 2,9 |
|  |  | Women | 31 | 8,7 ± 2,1 | 6,8 ± 2,8 | 2,7 ± 1,9 | 4,3 ± 4,3 | 4,9 ± 3,1 | 4,9 ± 2,9 | 4,8 ± 2,5 | 4,8 ± 1,6 | 4,6 ± 3,5 | 7,3 ± 3 |

**Supplementary Table S4.** Pearson’s correlation coefficients between the urinary levels of 5-(hydroxymethyl-2-furoyl)glycine (5-HMFG), 2-furoylglycine (2-FG) and other metabolites related to coffee intake.

|  | M0 | | M6 | | M12 | |
| --- | --- | --- | --- | --- | --- | --- |
|  | 5-HMFG | 2-FG | 5-HMFG | 2-FG | 5-HMFG | 2-FG |
| 2-FG | 0.72 | - | 0.74 | - | 0.72 | - |
| Caffeine | 0.32 | 0.38 | 0.41 | 0.37 | 0.37 | 0.40 |
| Paraxanthine | 0.39 | 0.43 | 0.41 | 0.42 | 0.36 | 0.43 |
| 1-Methylxanthine | 0.43 | 0.47 | 0.48 | 0.47 | 0.35 | 0.43 |
| 3-Methylxanthine | 0.56 | 0.46 | 0.45 | 0.38 | 0.40 | 0.36 |
| 1-Methyluric acid | 0.50 | 0.48 | 0.55 | 0.49 | 0.46 | 0.45 |
| 1,7-Dimethyluric acid | 0.35 | 0.41 | 0.37 | 0.36 | 0.30 | 0.39 |
| Cyclo(Leucyl-Proline) | 0.29 | 0.45 | 0.33 | 0.43 | 0.33 | 0.53 |
| Cyclo(Prolyl-Valine) | 0.37 | 0.50 | 0.38 | 0.47 | 0.44 | 0.63 |
| Trigonelline | 0.35 | 0.49 | 0.40 | 0.56 | 0.38 | 0.50 |
| N-Methylpyridinium | 0.27 | 0.46 | 0.30 | 0.43 | 0.31 | 0.55 |

**Supplementary Figure S1.** Flow chart of participants in the study and samples available for analysis at each study time point (M0, M6 and M12).
